# Power limits for microbial life

**DOI:** 10.3389/fmicb.2015.00718

**Published:** 2015-07-15

**Authors:** Douglas E. LaRowe, Jan P. Amend

**Affiliations:** ^1^Department of Earth Sciences, University of Southern California, Los AngelesCA, USA; ^2^Department of Biological Sciences, University of Southern California, Los AngelesCA, USA

**Keywords:** limits to life, thermodynamics, biogeochemistry, microbial ecology, organic matter degradation, modeling, bioenergetics, marine sediments

## Abstract

To better understand the origin, evolution, and extent of life, we seek to determine the minimum flux of energy needed for organisms to remain viable. Despite the difficulties associated with direct measurement of the power limits for life, it is possible to use existing data and models to constrain the minimum flux of energy required to sustain microorganisms. Here, a we apply a bioenergetic model to a well characterized marine sedimentary environment in order to quantify the amount of power organisms use in an ultralow-energy setting. In particular, we show a direct link between power consumption in this environment and the amount of biomass (cells cm^-3^) found in it. The power supply resulting from the aerobic degradation of particular organic carbon (POC) at IODP Site U1370 in the South Pacific Gyre is between ∼10^-12^ and 10^-16^ W cm^-3^. The rates of POC degradation are calculated using a continuum model while Gibbs energies have been computed using geochemical data describing the sediment as a function of depth. Although laboratory-determined values of maintenance power do a poor job of representing the amount of biomass in U1370 sediments, the number of cells per cm^-3^ can be well-captured using a maintenance power, 190 zW cell^-1^, two orders of magnitude lower than the lowest value reported in the literature. In addition, we have combined cell counts and calculated power supplies to determine that, on average, the microorganisms at Site U1370 require 50–3500 zW cell^-1^, with most values under ∼300 zW cell^-1^. Furthermore, we carried out an analysis of the absolute minimum power requirement for a single cell to remain viable to be on the order of 1 zW cell^-1^.

## Introduction

Over the last four decades, microorganisms have been found inhabiting environments with a broad range of physical and chemical conditions. Considerable effort has been channeled into finding the limits of temperature, pressure, pH, salinity, and other compositional and physical variables that hinder or prevent microbial activity (Rothschild and Mancinelli, 2001; Pikuta et al., 2007; Canganella and Wiegel, 2011; Colwell and D’Hondt, 2013; Takai et al., 2014). The attention focused on so-called extremophiles has not only buoyed the biotechnology industry (Wilson and Brimble, 2008), but has informed theories about the origin, evolution and extent of life, and has raised questions about the magnitude to which microorganisms modify geochemical processes. However, of all the variables that have been investigated as limiting life, one of the most fundamental has received much less attention: energy (Amend and Teske, 2005; Hoehler and Jørgensen, 2013) and the rate at which it is needed, power (LaRowe and Amend, 2015).

Virtually every aspect of microbial behavior requires energy supplied at a rate sufficient to meet power demands (Morowicz, 1968). This includes typical functions such as growth; nutrient uptake; motility; extracellular enzyme; and other biomolecular excretion; synthesis of external structures such as stalks, sheaths, tubes, and extracellular polymeric saccharides and changes in stored nutrients. In addition, microorganisms sometimes must use energy to combat environmental stressors such as desiccation, osmotic pressure, radiation, nutrient deficiency, elevated concentrations of toxins, an unfavorable, or oscillating redox state, asymbiosis, viruses, and predation. The response to these and other contingencies is limited by the genomic potential of the organism but also whether or not there is a sufficient flux of energy available to overcome them.

Although it is now possible to calculate the energy required to synthesize a great variety of biomolecules in any type of environment [see review by Amend et al. (2013), including energy transducing biomolecules such as ATP and NAD(P) (LaRowe and Helgeson, 2006, 2007], there are a number of uncertainties associated with the quantification of the power required to mount a response to environmental stresses. This is due to the variable amounts of biomolecular synthesis/repair, active pumping, and/or motility that might be required to combat any particular combination of selection pressures. For instance, in response to changing temperatures, many organism manipulate their lipid compositions to alter the rigidity of their cell membranes (Koga, 2012). This requires a series of steps including a mechanism for registering temperature, the expression of the proper genes, the actual synthesis of the desired lipids and other regulatory responses to ensure that the modifications promote survival.

Quantifying the energetic cost of each of these steps and the timescale over which microbial action is needed – and an unknown number of responses to other environmental stresses – is not currently achievable. It is, however, generally possible to quantify the amount of Gibbs energy (J mol^-1^) available in an environment if sufficient geochemical data are available (e.g., LaRowe et al., 2008, 2014; Shock et al., 2010; Amend et al., 2011; LaRowe and Amend, 2014; Osburn et al., 2014; Teske et al., 2014; Price et al., 2015). Typically, the required data include concentrations of major ions, electron donors and acceptors and any other chemical species that appear in reactions describing potential catabolic processes. This available energy can then be combined with measured or inferred reaction rates (mol s^-1^) to compute the amount of power (J s^-1^ or W) that is available in a particular environment (LaRowe and Amend, 2015). The resulting power supply is comparable to microbial maintenance power, the amount of power microorganisms expend that does not result in growth, in order to assess the carrying capacity of a given environment. Values of maintenance power do not directly reflect how much energy an organism is using as a response to stresses; instead, they account for the power that microorganisms use to carry out closely related tasks such as motility, changes in stored polymeric carbon, osmoregulation, extracellular secretions, defense against chemical stress, shifts in metabolic pathways, and the synthesis and turnover of macromolecules such as proteins and RNA (van Bodegom, 2007). In this way, maintenance power is a summary of the power that microorganisms use in order to keep the cell viable and ready to take advantage of opportunities.

The goal of this communication is to demonstrate a technique for determining how power constrains microbial activity, biomass and, ultimately, biogeochemical processes with the larger endgame of deciphering the power limits of life. This is done by categorizing microbial activity into three broad physiologic states – growth, maintenance, and decay. Although there are no clearly defined limits on the energy levels that demark the boundaries between each of these states, the amount of energy available in an environment can be compared to the number of cells in it to begin to understand how energy availability limits life. Marine sediments are an attractive test case for this approach because they comprise a broad spectrum of energy levels and varying amounts of biomass, and they are one of Earth’s largest habitats.

The lower power limits for life should not be confused with mechanistic arguments that have been made regarding microbial activity and energy minima (see Harder, 1997). Essentially, it has been proposed that microorganisms require a minimum amount of Gibbs energy from a catabolic reaction, Δ*G*_min_, due to the energetics of ATP synthesis, somewhere between -12 and -18 kJ per mole of reaction turnover (Schink, 1997; Hoehler, 2004). This idea has been formalized in kinetic models that represent reaction rates as a function of the energy available from catabolism, e.g., Jin and Bethke (2003). Most of these thermodynamic rate-limiting models stipulate that once the Gibbs energy of a particular catabolic reaction dip beneath a given minimum, microorganisms can no longer catalyze the reaction to gain energy (see LaRowe et al., 2012 for an overview). Within this paradigm, there is a minimum microbial energy demand that is equal to Δ*G*_min_. The approach taken in the current communication is that microbial activity is a function of the *rate* at which energy is made available and consumed, i.e., power. After all, 12 kJ could be seen as a large flux of energy if it is consumed in a second (12,000 W), or very little if consumed over the course of 100 years (0.000004 W). Microbial activity levels would be correspondingly divergent.

## Materials and Methods

LaRowe and Amend (2015) developed a model that directly relates power availability to microbial population dynamics. Within the constraints of this model, a microbial population should remain constant when the amount of power available in its environment is equal to the maintenance power of the community. Stated another way, the number of cells, *B*, that can be supported by a given power supply due to a particular catabolic reaction, *P*_s_, is related to the maintenance power demand, *P*_d_, through

(1) $$B=\frac{P_{s}}{P_{d}}$$

Because the number of microbial cells in marine sediments is typically given in units of cells cm^-3^, the units for *P*_s_ in Eq. (1) are W cm^-3^ and those for *P*_d_ are W cell^-1^. If the power supply is less than the collective maintenance demand, the number of cells in a population should decrease. Population decline should then persist until power supply and demand are equal. By combining Eq. (1) with the power supplied from a defined set of catabolic reactions, the potential number of active microbial cells in an environment can be estimated.

### Power Demand

The amount of power that microorganisms require depends on what physiologic state they are in. For example, growing microorganisms require far more power than those that are simply preventing their amino acids from becoming a lethal racemic mixture. Because this communication is focused on the lower limits of microbial activity, only the power states associated with maintenance activities are considered here. Although there is not an *a priori* method for calculating the amount of energy that microorganisms use at a given rate to maintain viability, a recent compilation of microbial maintenance powers shows that in the laboratory, microorganisms require 0.019–4700 × 10^-15^ J s^-1^ cell^-1^ (LaRowe and Amend, 2015). It should be noted that when these values are determined, maintenance refers to the power that microorganisms use that does not result in growth *while they are growing*. In natural settings, maintenance powers are likely much smaller than the above stated values since laboratory measurements of power must be conducted at relatively high levels in order to be observable in a reasonable time frame (Hoehler and Jørgensen, 2013). Hoehler and Jørgensen (2013) refer to the maintenance power of organisms that are not growing as basal maintenance power, which is the relevant metabolic state for microorganisms in the oligotrophic marine sediments considered in this study. Furthermore, it should be noted that values of maintenance power are average values determined for populations. Any one particular organism may be demanding power at a greater or lesser rate.

### Power Supply

The power available for microbial activity in a particular setting is the sum of the power resulting from all of the potential catabolic reactions in an environment. The power supplied by each catabolic reaction being catalyzed is the product of the Gibbs energy of the reaction, Δ*G_r_*, multiplied by the rate, *r*, at which these reactions are catalyzed:

(2) $$P_{s}=\Delta G_{r}\cdot r$$

Calculated values of *P_s_* in different natural settings differs by as much as 12 orders of magnitude (LaRowe and Amend, 2015), due mostly to the variability of catabolic rates (Orcutt et al., 2013).

#### Gibbs Energies

Values of Δ*G_r_* are calculated using

(3) $$\Delta G_{r}=\Delta G_{r}^{o}+RT\ln Q_{r}$$

where ΔG_r_^o^ and *Q*_r_ refer to the standard molal Gibbs energy and the reaction quotient of the indicated reaction, respectively, *R* represents the gas constant, and *T* denotes temperature in Kelvin. Here, values of ΔG_r_^o^ are calculated using the revised-HKF equations of state (Helgeson et al., 1981; Tanger and Helgeson, 1988; Shock et al., 1992), the SUPCRT92 software package (Johnson et al., 1992), and thermodynamic data taken from (Shock and Helgeson, 1988, 1990; Shock et al., 1989; Sverjensky et al., 1997; Schulte et al., 2001; Richard, 2006). Values of *Q_r_* are calculated using

(4) $$Q_{r}=\Pi \;a_{i}^{v_{i}},$$

where *a_i_* stands for the activity of the *i*th species and *v_i_* corresponds to the stoichiometric coefficient of the *i*th species in the reaction of interest. Molalities of the *i*th species, *m_i_*, were converted into activities using individual activity coefficients of the *i*th species (γ*_i_*),

(5) $$a_{i}=m_{i}\gamma_{i}.$$

Values of γ*_i_* were in turn computed as a function of temperature and ionic strength using an extended version of the Debye-Hückel equation (Helgeson, 1969).

Values of Δ*G_r_* calculated for reactions in natural environments generally range from endergonic (Δ*G_r_* > 0) to about -120 kJ (mol e^-^)^-1^ (e.g., Amend et al., 2003; Shock et al., 2010; Osburn et al., 2014).

#### Reaction Rates

Like the Gibbs energy function, the rates of microbially catalyzed catabolic reactions are also a function of numerous chemical and physical variables. However, unlike thermodynamic formulations, the many equations that describe the kinetics of various chemical reactions are path dependent (Lasaga, 1981). This means that there are no general equations that link environmental conditions to the rates of biologically mediated reactions. As a result, the rates of microbially catalyzed reactions in marine sediments are commonly deduced from the application of models to geochemical data (e.g., Van Cappellen and Wang, 1996; Regnier et al., 2011).

The reactive continuum model (RCM), proposed by (Boudreau and Ruddick, 1991) is used here to compute the rate of reactions supplying energy to marine microbiological communities from the degradation of particulate organic carbon (POC). Continuum models not only capture the observation that POC degradability decreases with depth (Middelburg and Meyesman, 2007), but are well suited for describing the spatial and temporal dynamics of organic carbon in marine sediments that are not directly observable (Arndt et al., 2013). Of the many model types that have been used to quantify the degradation of POC (for a review see Arndt et al., 2013), the Boudreau and Ruddick RCM captures the observed degradation of organic matter (OM) because it conceptually represents the dynamics of OM degradation in marine sediments (Aller and Blair, 2004) within a convenient mathematical form (Arndt et al., 2013).

From a conceptual perspective, the Boudreau and Ruddick RCM treats POC as a large collection of many organic compounds whose reactivities form a continuous gamma distribution – from relatively labile to very recalcitrant (see Davis, 1963 for a description of the gamma function). The initial distribution of these compounds at a particular location and how they react as a function of time can vary. That is, in a given cm^3^ of sediment, the POC in it is represented as containing a range of different types of organic compounds whose ability to be consumed by microorganisms varies as a function of the age of the sediment and the distribution of compounds that were first deposited. In order to use the continuum model, the amount of particulate OM deposited at the sediment-water interface, SWI, and an age model for the sediments must be known or assumed. In addition, there are two free parameters that must be estimated that describe the nature of the OM that reaches the SWI. These parameters can be determined for a particular setting by fitting the measured amounts of POC in sediments to a function consistent with the RCM (see below).

The degradation rate of POC, *R_POC_*, (mol cm^-3^ year^-1^) is given by

(6) $$R_{POC}=\frac{v}{\left(a+age(z)\right)}POC_{0}{\left(\frac{a}{a+age(z)}\right)}^{v}$$

where ν designates how the compounds are distributed in a sample (unitless), *a* corresponds to the apparent initial age of the POC (years), *age*(*z*) indicates the age of the sediment (years) and *POC*_0_ stands for the amount of POC at the SWI (mol cm^-3^). For a constant value of ν, high values of the *a* parameter indicate an older assemblages of organic carbon consisting of compounds that are more resistant to degradation. Similarly, lower values of ν indicate that the mixture of organic compounds is dominated by refractory compounds not easily consumed by microorganisms. The POC deposited in open ocean sediments under oligotrophic water with long transit times to the ocean floor therefore would typically be characterized as having large values of *a* and small values of ν, as compared to POC deposited with sediments near productive ocean margins.

Values of ν and *a* were determined for the South Pacific Gyre IODP Site U1370 (41°51.1289′S, 153°6.3799′W for hole 1370A) by carrying out a non-linear regression of POC concentrations as a function of depth, *POC_z_*, with

(7) $$POC_{z}=POC_{0}{\left(\frac{a}{a+age(z)}\right)}^{v}$$

(see below for site description). The age of sediment layers as a function of depth, *age(z)*, were calculated by assuming steady state compaction and an exponential decrease of porosity with depth (Berner, 1980):

(8) $$age(z)=\frac{z+\frac{\phi_{0}}{c_{0}}\left(e^{-c_{0}\cdot \;z}-1\right)}{\omega_{0\;}\left(1-\phi_{0}\right)}$$

where ϕ_0_ and ω_0_ refer to the porosity and sedimentation rate at the SWI and *c*_0_ corresponds to the compaction length scale. The value of ϕ_0_ (0.87) was taken from D’Hondt et al. (2011), whereas those for ω_0_ (10^-6^ m year^-1^) and *c*_0_ (0.00085 m^-1^) were taken from (Burwicz et al., 2011). For reference, the sedimentation rate at Site SP-15, which is relatively close to Site U1370 (40°00.531′S, 154°2.601′W), has been dated to be 0.8 × 10^-6^ m year^-1^ over the last 17.5 Myrs (Stancin et al., 2008).

## Results

**FIGURE 1:**
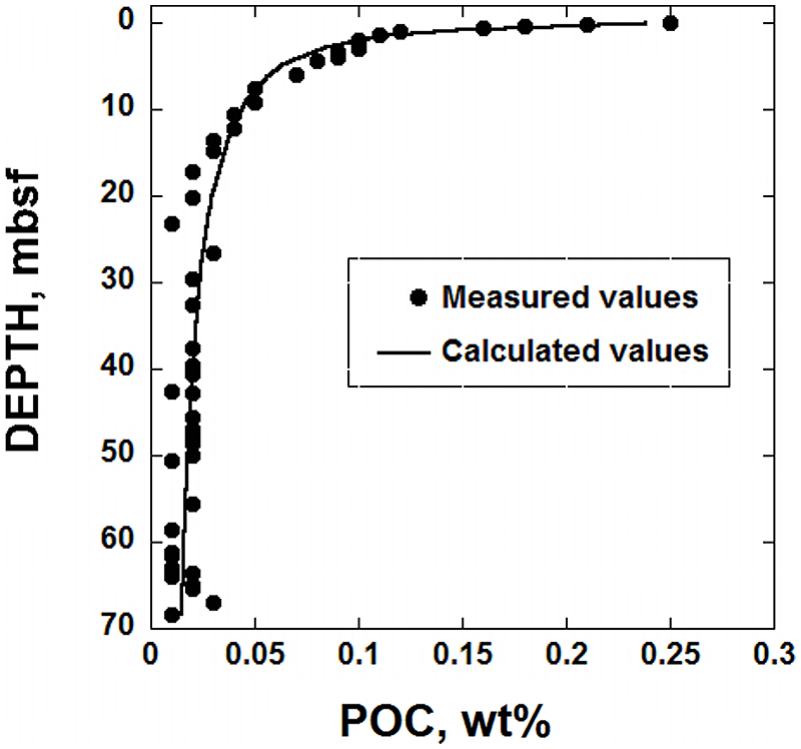
**Particulate organic carbon (POC), as a function of depth in sediments taken from IODP hole U1370 (South Pacific Gyre).** The circular symbols stand for measured POC in units of dry weight percent and the line represents a calculated fit to these data using the reactive continuum model (RCM) proposed by Boudreau and Ruddick (1991).

**FIGURE 2:**
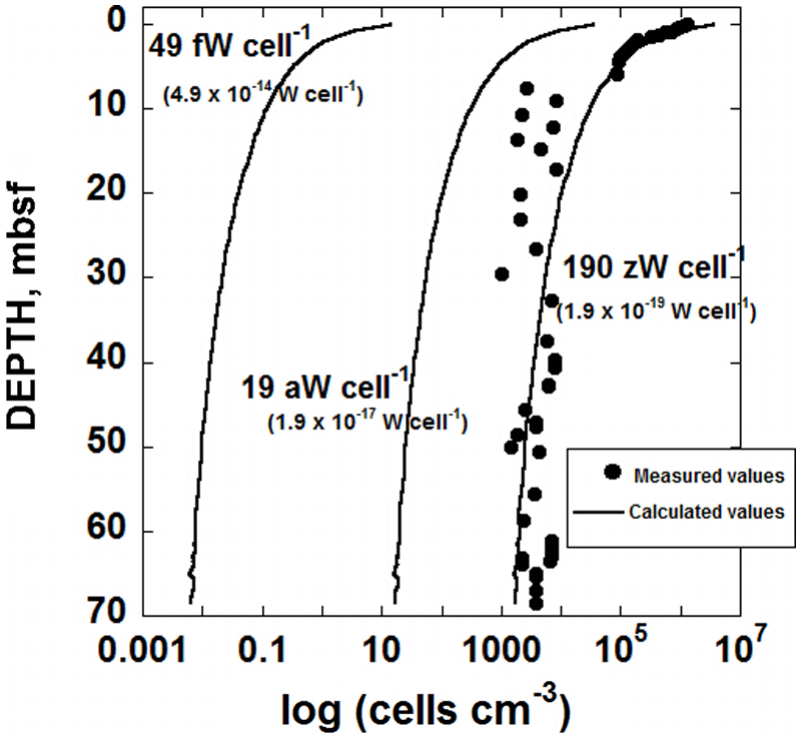
**Distribution of biomass in sediments taken from IODP hole U1370 (South Pacific Gyre) as a function of depth in units of cells cm^-3^.** The circular symbols refer to cell counts (D’Hondt et al., 2015) and the lines correspond to calculated cell densities (cells cm^-3^) using three different values of cell-specific maintenance powers, *P_d_*, indicating an average of values taken from the literature for aerobic carbon oxidation, *P_d_* = 49 fW cell^-1^ (4.9 × 10^-14^ W cell^-1^), the lowest value of *P_d_* reported in the literature, *P_d_* = 19 aW cell^-1^ (1.9 × 10^-17^ W cell^-1^) and a values of *P_d_* two orders of magnitude lower than this low, *P_d_* = 190 zW cell^-1^ (1.9 × 10^-19^ W cell^-1^).

The RCM is used with Gibbs energy calculations to describe the amount of power produced by microorganisms in marine sediment under the South Pacific Gyre. The sediments considered in this study are characterized by low sedimentation rates (∼93 cm/Myr) and are under 5074 m of water, leading to very low levels of OM fluxes to the SWI (D’Hondt et al., 2009). Because the delivery rate of OM is so slow, the concentration of it is low, and decreases with depth due to microbial activity. For example, at 6 cm below the SWI, POC is 0.25% of the sediment dry weight and at 27 m below SWI, it’s less than 0.03% (D’Hondt et al., 2011). The distribution of POC at Site U1370 is shown in **Figure 1** (circular symbols). It should be noted that the reported concentrations of POC from the deepest half of the sediments column show very little variation with depth. This could mean that there is no microbial degradation of OM deeper than ∼30 m below seafloor, but it should be kept in mind that the analytical procedures used to measured TOC are not sensitive enough to discern fine-scale changes in TOC (see below for Discussion). As in many sedimentary environments, there is a sharp decrease in POC concentration in the uppermost portion of the sediment column, followed by a much smaller decrease as a function of depth. If the amount of POC arriving at the SWI is roughly constant over time, this pattern can be explained by relatively rapid microbial degradation of POC soon after deposition, with consumption rates slowing considerably with time and possibly stopping altogether at >30 mbsf. Although the decreasing reactivity of POC is sometimes due to low concentrations of terminal electron acceptors (e.g., Van Cappellen and Gaillard, 1996; Boudreau, 1997; Thullner et al., 2007), this is not the case in hole U1370; O_2_ concentrations throughout hole U1370 are >7 μM (D’Hondt et al., 2015). Therefore, the persistence of OM is likely due to a combination of factors including the increasing proportion of recalcitrant organic compounds constituting the POC. The slower rates of POC degradation with depth correspond with fewer cells in the sediment column as well. The cell counts for IODP Site U1370 (D’Hondt et al., 2015), shown in **Figure 2**, illustrate this pattern – relatively high cell counts where the POC is high, and much lower cell counts where POC is low. In fact, at >6 mbsf, cell counts are <10^4^ cells cm^-3^. In order to establish a quantitative link between the rate of energy supply from POC in this environment and the number of cells found in SPG sediments, the RCM is applied here in conjunction with the bioenergetic model proposed by LaRowe and Amend (2015).

Using the data shown in **Figure 1**, an assumed constant sedimentation rate and Eqs (7 and 8), the best-fit parameters required to use the RCM at SPG Site U1370 were determined to be *POC*_0_ = 0.255 wt.%, *a* = 445,990 years and *v* = 0.5566 (see below for uncertainties). It can be seen by the curve in **Figure 1** that these parameters and Eq. (7) closely capture the distribution of POC in hole U1370. In order to assess how much energy is available from this OM, the reported POC concentration in units of dry weight percent must be converted into units that can be quantitatively scaled to how biomass is often presented in sediments, in units of cells cm^-3^. As a result, the POC data shown in **Figure 1** were converted into moles of OM per cm^3^ of sediment. These values were calculated using porosity and bulk density data for hole U1370 (D’Hondt et al., 2011) and an assumed chemical formula of OM equal to C_27_H_28_O_7_ (see Richard, 2006), a complex organic compound that has nearly the same average nominal oxidation state of carbon (NOSC), -0.52, as OM in other marine sedimentary environments (personal communication, B. Kiel-Reese). The resulting values of OM per cm^3^ of SPG sediment are between about 10^-6^ and 10^-7^ moles of POC.

**FIGURE 3:**
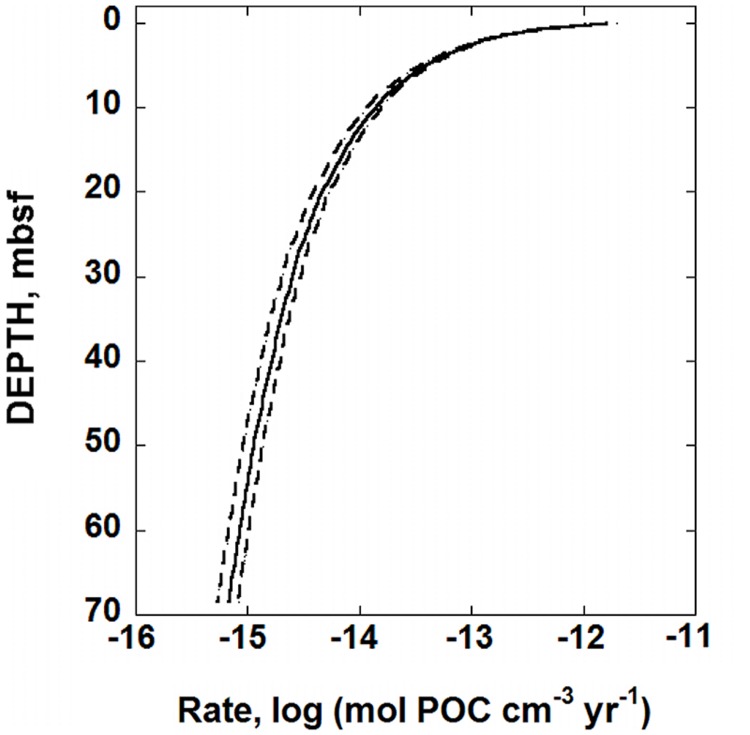
**Calculated rates of POC, degradation in sediments from IODP hole U1370 (South Pacific Gyre) as a function of depth in units of moles of POC cm^-3^ year^-1^.** The solid line was generated using the best-fit values of the RCM, Eq. (7), to the POC data shown in **Figure 1**. The dashed lines represent the maximum uncertainties of POC degradation rates. The RCM parameters that were used to produce these lines are those that produce the most variation in calculated rates of POC degradation, but are within the range of uncertainties for each parameter: *POC*_0_ = 0.2648 wt.%, *a* = 358,158 years and *v* = 0.5871 (dashed line on the right) and *POC*_0_ = 0.2462 wt.%, *a* = 533,822 years, and *v* = 0.5260 (dashed line on the left).

The OM degradation parameters determined for IODP hole U1370 can be combined with Eq. (6) to calculate the rate of POC degradation, mol POC cm^-3^ year^-1^. The results are shown in **Figure 3**. The solid line in this figure was calculated using the best-fit RCM parameters, and the dashed lines reflect the uncertainties associated with using Eq. (7) to regress the POC data (see below). At the top of the sediment column, less than 10^-11^ mol POC cm^-3^ year^-1^ are consumed by microbial activity. At about 12 mbsf, the rate of POC degradation decreases to ∼10^-14^ mol POC cm^-3^ year^-1^. At the bottom of the sediment core, the calculated POC degradation rate using the best-fit scenario is a meager 6.8 × 10^-15^ mol POC cm^-3^ year^-1^. Again, it should be noted that the distribution of POC shown in the bottom half of the sediment column in **Figure 1**, does not show much, if any, variation with depth. Therefore, the rates calculated for this portion of the sediment column could be an artifact of the underlying, assumed gamma distribution characterizing the reactivity of OM in the RCM.

The calculated rates of POC degradation can in turn be combined with Gibbs energies of organic carbon degradation to calculate the power dissipated by microorganisms in SPG sediments. Using Eqs (3–5), values of Δ*G_r_* of POC degradation are calculated for

(9) $$C_{27}H_{28}O_{7}+{13H}_{2}O+30.{5O}_{2\;\;\;}\to {27HCO}_{3}^{-}+{27H}^{+}.$$

**FIGURE 4:**
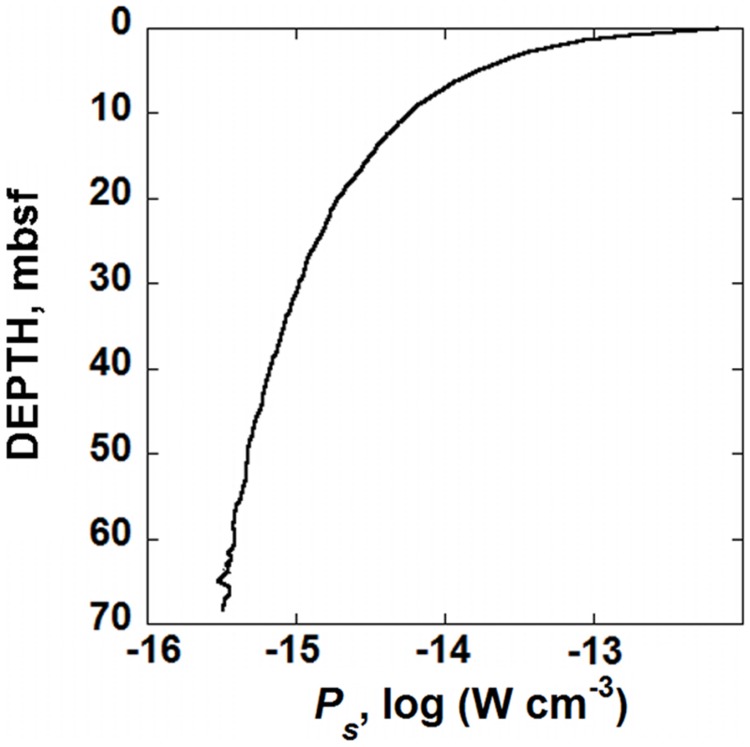
**Calculated power supply, *P_s_*, from the microbial degradation of POC, in sediments from IODP hole U1370 (South Pacific Gyre) as a function of depth in units of W cm^-3^**.

The values of *Q* required to compute Δ*G_r_* were calculated using down-core concentrations of O_2_, HCO_3_^-^, and H^+^ reported for Site U1370 (D’Hondt et al., 2011). The values of Δ*G_r_* for Rxn. (9) were then used in conjunction with the rates of POC shown in **Figure 3** and Eq. (2) to calculate the amount of power available for organisms catalyzing the degradation of OM in SPG sediments. The resulting power supply, *P_s_* (in W cm^-3^), is shown as a function of depth in **Figure 4**. Following the same pattern as the rates of POC degradation, **Figure 3**, the number of Joules available per second per cubic centimeter of sediment is relatively large near the SWI, 1.6 × 10^-12^ W cm^-3^, but drops rapidly with depth. At 20 mbsf, the decrease in power yield slows to 1.9 × 10^-15^ W cm^-3^ and at 68 mbsf, the calculated value of *P_s_* is about 3.3 × 10^-16^ W cm^-3^. The change in power available from POC is mostly due to the changing rates of degradation, rather than the changing Gibbs energies of Rxn (9). That is, the percent difference between the maximum and minimum values of Gibbs energies throughout the sediment column is less than 8% whereas POC degradation rates change by over three orders of magnitude.

In order to estimate how many microorganisms can be sustained by the power supply shown in **Figure 4**, the amount of power that microorganisms in this environment use must be determined. As noted above, the amount of power that a microorganism demands is a function of its physiological state. The classical view of microbial physiology categorizes microbial activity as being in the lag, exponential, stationary, or death phases. However, these stages are based on laboratory growth experiments of culturable organisms over very short time scales. In most environments, especially low energy settings, the vast majority of microorganisms are apparently not culturable and might be growing extremely slowly, perhaps doubling (or replacing biomass) once every several-1000 years (Jørgensen and Boetius, 2007; Jørgensen, 2011). The perspective taken in this communication is that being in a state of maintenance refers to the steady state condition in which cells have enough power available to carry out tasks that are required to ensure viability, akin to the basal maintenance power noted by Hoehler and Jørgensen (2013).

A demonstration of the predictive capability of laboratory-determined maintenance powers is shown in **Figure 2**. Here, the number of cells that can be maintained by the power that is available from the aerobic degradation of POC is shown for a variety of different cell-specific maintenance powers. For reference, the sediment cell counts from IODP hole U1370 are presented as circular symbols. Each of the lines in **Figure 2** represents calculated numbers of cells per cm^-3^ in SPG sediments. These values were computed using the power supply from POC degradation as determined above (**Figure 4**), a rearranged version of Eq. (1) and three different values of cell-specific maintenance powers, *P_d_*. The left-most line in **Figure 2** was generated with *P_d_* = 49 fW cell^-1^ (fW, femtowatt, 10^-15^ W), the lowest maintenance power reported in the literature for aerobic heterotrophy (Tijhuis et al., 1993). The middle curve in **Figure 2** was generated using the overall lowest cell-specific maintenance power reported in the literature, 19 aW cell^-1^ (aW, attowatt, 10^-18^ W; Marschall et al., 2010), and the right-most curve was made using a *P_d_* two orders of magnitude lower than this, following the suggestion by Hoehler and Jørgensen (2013) that in nature, basal maintenance powers could be two of more orders of magnitude lower than maintenance powers measured in the laboratory. It can be seen in **Figure 2** that the biomass (cell cm^-3^) curve corresponding to the lowest maintenance powers considered, 190 zW cell^-1^ (zW, zeptowatt, 10^-21^ W), most closely matches the cell counts measured in SPG Site U1370. The lowest laboratory-based values of *P_d_*, 49 fW cell^-1^, for aerobic heterotrophy is the furthest from capturing the observed distribution of biomass in hole U1370, and even the lowest reported value of *P_d_* (19 aW cell^-1^) is orders of magnitude from predicting the number of cells in this sedimentary setting.

## Discussion

The work described above is a quantitative effort to relate the number of microorganisms in a very low energy environment to its chemical composition. The degradation rate of POC and the microbial power supply calculations that were required to carry this out rely on the combination of a number of data sets, assumptions, and calculations. As with any numerical modeling, there are caveats and associated uncertainties. The following sections explore issues associated with the energetics of OM degradation, the uncertainties of using the RCM and other catabolic reactions that yield energy in SPG sediments. Finally, the last part of this communication explores what the fundamental power requirements are for microbial life.

### Particulate Organic Carbon

As noted above, the lack of a gradient in measured concentrations of POC with depth (**Figure 1**) does not support the calculated rates of its degradation below ∼30 mbsf (**Figure 3**). This could be due to a lack of microbial activity in the deeper portions of the sediment column, or to the inability of the methods used to measure POC concentrations to detect minute changes in POC concentration resulting from the very low rates of POC degradation. However, there are several lines of evidence supporting the notion that microorganisms are degrading OM at IODP Site U1370. To begin with, although the POC that exists deep in SPG sediments is likely quite resistant to microbial breakdown, this does not mean that this OM is not biologically active. In fact, it has been shown that kerogens and other relatively recalcitrant molecularly uncharcterizable OM is indeed slowly transformed by microorganisms (Petsch et al., 2000; Henrichs, 2005; Moodley et al., 2005; Burdige, 2007) In addition, it has also been shown that microorganisms from deep marine sediments as old as 16 Myrs have ribosomes and are thus alive and active (Schippers et al., 2005). Morono et al. (2011) demonstrated that a majority of micorbes taken from sediments 219 mbsf were able to take up isotopically labeled C and N. Furthermore, virus counts in numerous marine sedimentary environments have shown that microbial cells are viable in, among others, SPG sediments down to about 55 Myrs-old horizons (Engelhardt et al., 2014). Finally, and perhaps most importantly, a recent study has reported O_2_ reduction rates and microbial cell counts at IODP Site U1370 that support the contention that POC is degraded throughout the sediment column.

At the SPG Site U1370, O_2_ is present throughout the sediment column all the way to basement rock, but decreasing in concentration with depth (D’Hondt et al., 2009, 2015). D’Hondt et al. (2015) claim that the decrease in O_2_ at Site U1370 is due to microorganisms oxidizing OM. In particular, they demonstrate that oxygen is being consumed in sediments from 37.5 to 68 mbsf at a rate of 3.3 × 10^-13^mol O_2_ cm^-3^ year^-1^. The rates of POC degradation calculated here using the best-fit RCM from 37.5 to 68 mbsf are 1.8 × 10^-15^ to 6.7 × 10^-16^mol POC cm^-3^ year^-1^. Using the stoichiometry of Reaction (9), these rates of POC degradation translate into O_2_ reduction rates of 5.5 × 10^-14^ and 2.0 × 10^-14^mol O_2_ cm^-3^ year^-1^, about an order of magnitude less than the O_2_ reduction rate reported by D’Hondt et al. (2015). It is intriguing that two very different methods and data sets come within an order of magnitude predicting the same rates of microbial activity in an extremely low power environment. At the very least, the rates of O_2_ reduction determined by D’Hondt et al. (2015) are more than sufficient to account for the rates of POC degradation shown in **Figure 3**. From a conservative perspective, it could be the case that there is active microbial degradation of OM only in the top ∼30 m of the sediment column since the amount of POC beneath this depth does not decrease very much (**Figure 1**). However, given the existence of microbial cells throughout the sediment column (**Figure 2**), the disappearance of O_2_ at rates consistent with the calculated POC degradation rates and the evidence for active microbial communities deep in marine sediments presented above, there is sufficient reason to conclude that OM degradation could very well be occurring at very low rates deep in the sediments of the South Pacific Gyre.

The use of a single organic compound, C_27_H_28_O_7_ (Reaction 9), to represent POC in marine sediments is a gross simplification of the likely organic compounds in marine sediments (Burdige, 2007). Although the uncertainty of not knowing which organic compounds to use in the reactions that describe POC degradation can have an energetic consequence of up to 23 kJ (mol e^-^)^-1^, the fact that O_2_ is the likely oxidant in U1370 dominates the energetics of POC degradation (LaRowe and Van Cappellen, 2011). For instance, Δ*G_r_* for Rxn. (9) in the topmost portion of U1370 is -13,310 kJ mol POC^-1^. If, for example, acetate was used to represent POC, Δ*G_r_* would be ^-^903 kJ mol POC^-1^. At first glance, this seems like a large difference. However, since acetate has a much smaller molecular weight than C_27_H_28_O_7_, the conversion from wt. % POC cm^-3^ to mol POC cm^-3^ yields a much larger number of moles of POC. As a result, the power supplied by Rxn. (9) at 6 cm below the SWI is 6.75 × 10^-13^ W cm^-3^, and for the analogous reaction containing acetate instead of C_27_H_28_O_7_, it’s 3.49 × 10^-13^ W cm^-3^. In the absence of more specific information on the concentrations and types of organic compounds in U1370, using a large suite of organic compounds only introduces more uncertainty.

One of the largest uncertainties associated with using the RCM to represent the rates of POC degradation is that the sedimentation rate, and therefore the accumulation rate of organic carbon to the sediment water interface, is assumed to be constant. We assumed that the sedimentation rate is constant, and calculated it using the thickness of the sediment column and the age of the bedrock underlying Site U1370, ∼75 million years (D’Hondt et al., 2011, 2015). The actual rate of POC delivery to sediments at this location since the end of the Cretaceous is simply unknown. However, it should be noted that plate reconstructions show that the area surrounding Site U1370 has been far from land throughout this time period (Scotese et al., 1988).

**FIGURE 5:**
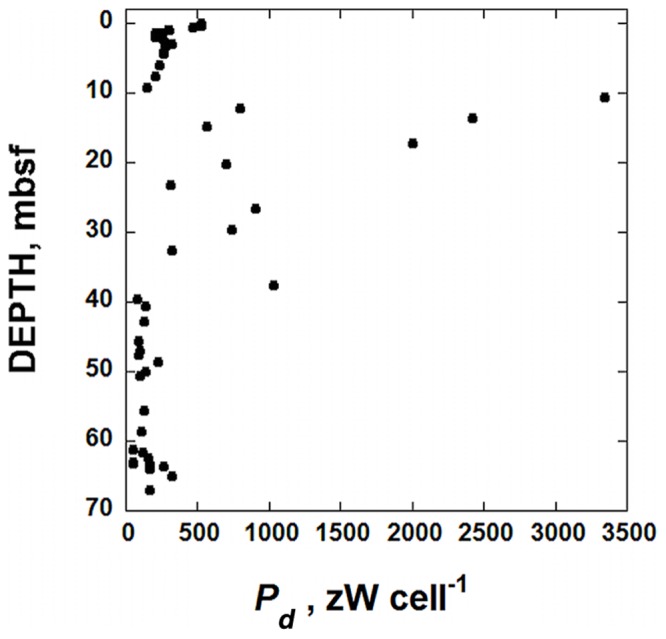
**Calculated values of cell-specific maintenance powers, *P_*d*_*, in sediments taken from IODP hole U1370 (South Pacific Gyre) as a function of depth in units of zW cell^-1^ (10^-21^ W cell^-1^)**.

Another source of uncertainty is the values of the RCM parameters associated with the fit of Eq. (7) to POC as a function of depth (**Figure 1**). Overall, the coefficient of determination, *R*^2^, for the fit shown in **Figure 1** is 0.978, and a plot of the residuals for this fit (not shown), indicate that there are no systematic over- or under-predicted values of POC. The randomness of the residuals and the high value of *R*^2^ strongly support the notion that the RCM closely captures the pattern of POC in the sediments at SPG Site 1370. Furthermore, due to the functional form of the fit, Eq. (7), the uncertainties of the RCM parameters are not equal. The best-fit values and their associated SE (1σ) are as follows: *POC*_0_ = 0.2554 ± 0.0093 wt.%, *a* = 445,990 ± 87,830 years, and *v* = 0.5566 ± 0.0305, ± 3.6, ± 20, and ± 5.5%, respectively. The two combinations of these RCM parameters that produce the most variation in calculated rates of POC degradation from the best-fit values, *POC*_0_ = 0.2648 wt.%, *a* = 358,158 years and *v* = 0.5871, and *POC*_0_ = 0.2462 wt.%, *a* = 533,822 years, and *v* = 0.5260, are also used with Eq. (6) to calculate rates of POC degradation at SPG Site U1370. The results are shown by the dashed lines in **Figure 3**, which bracket the best-fit rate, the solid line. The difference in calculated POC degradation rates generated from the best-fit RCM parameters and the combination of RCM parameters that result in the maximum variance from the best-fit scenario gradually expand with depth. The maximum percent difference between the best-fit calculated rate of POC degradation and that of the two alternative fits is 22.9% with an average percent difference of 15.6%. If these alternative rates of POC degradation are propagated through the calculations for power supply (**Figure 4**) and the number of cells that can be supported at various maintenance powers (**Figure 5**), then we would expect ∼16% difference from the RCM parameter uncertainties. However, it should be kept in mind that the alternative RCM parameter values used to generate the dashed lines in **Figure 3** are effectively the worst of the probable fits of Eq. (7) to the POC data.

### Bioenergetic Model

We have assumed that the organisms located near IODP Site U1370 are not growing, but are preserving their population size by obtaining energy at a rate that is equivalent to an average cell-specific basal maintenance power. If these SPG microorganisms are indeed maintaining themselves by oxidizing POC, then the maintenance powers taken from the literature are, as has been suggested (Hoehler and Jørgensen, 2013), far too high to explain the amount of biomass living in low-power environments. There are, however, other reasons why the literature-based values of *P_d_* used to generate two of the curves in **Figure 2** seem too high. One notion is that most of the cells in this environment could be intact but inactive. This is unlikely since the O_2_ concentration measured in these sediments decreases with depth (D’Hondt et al., 2009, 2011, 2015), suggesting that O_2_ is consumed in the sediment by microorganisms faster than it can diffuse in from the overlying water column. Another possibility is that the vast majority of cells in these sediments are not involved in POC degradation. It has been suggested that the knallgas reaction could be powering microbes in SPG sediments from hydrogen supplied by the radiolysis of water (D’Hondt et al., 2009). Similarly, an energy-density analysis has shown that the oxidation of Fe^2+^ and Mn^2+^ is a potential source of power at nearby SPG Site U1365 (LaRowe and Amend, 2014). Nonetheless, it is intriguing that the single-cell maintenance power of 190 zeptowatts, a number chosen simply by virtue of being exactly two orders of magnitude lower than the lowest reported maintenance power in the literature, closely predicts the number of cells that have been counted in SPG sediments. Finally, the possibility exists that the power supplied by POC degradation (and other reactions) is not meeting maintenance power demands and the microbial populations are actually shrinking at an extremely slow rate.

### Power Minimum for Microbial Life

The minimum amount of power required to sustain a microbial cell is not known, nor is the rate at which a population will shrink when the power supply is lower than the power demand from maintenance. Nonetheless, the results shown in **Figure 2** can be used in conjunction with other observations reported in the literature to estimate what the power minimum for life is.

When power supply is lower than power demand, the size of a microbial population will eventually decrease. This is due to the fact that all cells, no matter their physiological state or the prevailing environmental conditions, face stresses that will eventually require energy-demanding responses if the cells are to remain viable. Ultimately, this is due to the fact that all organic biomolecules are metastable. As a result, the rate at which a microbial population decreases depends on how low of a metabolic state the organisms can achieve and maintain, and the rate of biomolecular decay. An extremely low-power strategy used by some types of microorganism is to form cysts or endospores when environmental conditions become unfavorable. These long-term hibernation strategies, and other extremely low-power states that may exist in power-limited settings, have physiochemical limits such as the abiotic isomerization of amino acids, sugars, and membrane hydrocarbons, the depurination and depyrimidination of nucleotides and the deamination of amino acids and other amine-containing compounds. The abiotic rates of these processes are variable, but slow, and in the case of amino acid isomerization, depend on pH, temperature, whether the amino acid is free or in a polymer of a particular length and other factors (Wehmiller and Hare, 1971; Kvenvolden et al., 1973; Bada and Schroeder, 1975; Killops and Killops, 2005; Steen et al., 2013). Nonetheless, if spores and other virtually inactive cells do not have a mechanism and the energy to repair these molecular transformations, the damaged biomolecules will accumulate to a level that will prevent viability. Although it is known that microorganisms possess a vast array of isomerases and other enzymes that can repair these defects (Webb, 1992), the amount of power required to retain molecular-level viability is unknown and difficult to assess. One would need to know the rates at which all of these, and likely other, abiotic reactions occur in particular environmental contexts, the maximum number and combination of defective biomolecules that can be tolerated by various species and the amount of energy required to repair each of them.

Despite these difficulties, a back-of-the-envelope style determination of the power level required to prevent population decay is worth attempting since knowledge of this quantity is key to understanding how long microorganisms can persist in low-power environments. This can be accomplished by combining the lowest reported maintenance power in the literature, 19 aW or 1.9 × 10^-17^ J s^-1^ cell^-1^ (Marschall et al., 2010), with the estimate that the average metabolic rate required to prevent racemization of amino acids is about 200 times less power than that for maintenance in prokaryotes [this is extracted from the difference in the lines representing the temperature dependence of maintenance and amino-acid racemization prevention power, by Price and Sowers (2004), **Figure 1**]. This leads to a decay prevention power of 9.5 × 10^-20^ W cell^-1^, less than 1 eV s^-1^. For comparison, the dissociation enthalpy of chemical bonds typically ranges from about 1 to 10 eV (Tinoco et al., 1995). Obvious problems with this calculation include the fact that the anti-racemization line presented by Price and Sowers (2004) is based on rates of aspartic acid racemization in wet Siberian permafrost soils (i.e., Brinton et al., 2002) and that laboratory-measured maintenance powers might be two or more orders of magnitude higher than those in natural, low-energy settings – basal maintenance powers (Hoehler and Jørgensen, 2013). Taking this notion from Hoehler and Jørgensen, the anti-decay power stated above (9.5 × 10^-20^ W cell^-1^, 95 zW cell^-1^) would then be reduced to ∼1 zW cell^-1^. This is equivalent to about 3 × 10^-14^ J cell^-1^ year^-1^. On a molecular scale, acetate oxidation coupled to sulfate reduction under sedimentary conditions (*T* = 15°C, *P* = 250 bars, activities of sulfate, acetate, sulfide, and bicarbonate equal to 0.01, 10^-5^, 10^-6^, and 0.002, Δ*G_r_* = -70,409 J mol^-1^) translates to 1.4 × 10^-26^ mol acetate s^-1^ cell^-1^. In a year this is 4.3 × 10^-19^ mole acetate or 260,000 molecules. Given that eight electrons are transferred per mole of completely oxidized acetate, 2.1 × 10^6^ relatively low-energy electrons per year are required to prevent the molecular decay of one microbial cell.

The decay prevention power of 1 zW cell^-1^ can be compared to calculated cell-specific maintenance powers, *P_d_*, for microbes in IODP Site U1370. These values of *P_d_* can be calculated using the power supply from POC degradation (**Figure 3**), reported cells counts (**Figure 2**) and Eq. (1). The resulting cell-specific maintenance powers are shown in **Figure 5**. They range from just less than 3500 zW cell^-1^ to 49 zW cell^-1^ with most values below 500 zW cell^-1^. Røy et al. (2012) report that organisms in sediments under the oligotrophic North Pacific Gyre, which is very similar to the SPG, are respiring oxygen with organic carbon in deep sediments at rates down to 10^-18^ mol O_2_ cell^-1^ day^-1^. Using the same methods outlines above, this translates to about 5.3 × 10^-18^ W cell^-1^, or 5300 zW cell^-1^, slightly higher than the highest value calculated for SPG sediments. It could be the case that the estimate of 1 zW cell^-1^ to prevent microbial molecular decay is off by an order or magnitude or more, but it is intriguing that the calculated maintenance powers of the most power-limited cells in the sediment column at Site U1370 are only about one and half orders of magnitude larger than 1 zW cell^-1^. We do not claim that this is the absolute minimum amount of power required by microorganisms, but rather an attempt to rationally arrive at such a number. Naturally, the minimum power requirement for life is a function of the environmental surrounding. A warmer environment will have accelerated rates of molecular decay, and one subjected to solar radiation, predators, and more chemical and physical fluctuations than a quiescent marine sediment would surely dictate higher power requirements to prevent decay. At the very least, the analysis presented above is a quantitative effort to define the power limits to life.

## Conflict of Interest Statement

The authors declare that the research was conducted in the absence of any commercial or financial relationships that could be construed as a potential conflict of interest.
